# A review of *Diplommatina* species in eastern Thailand with the descriptions of five new species

**DOI:** 10.3897/BDJ.8.e57689

**Published:** 2020-10-06

**Authors:** Pongrat Dumrongrojwattana, Chanakarn Kamtuptim, Koraon Wongkamhaeng

**Affiliations:** 1 Department of Biology, Faculty of Science, Burapha University, Chon Buri, Thailand Department of Biology, Faculty of Science, Burapha University Chon Buri Thailand; 2 Kasetsart University, Bangkok, Thailand Kasetsart University Bangkok Thailand

**Keywords:** *
Diplommatina
*, Diplommatinidae, taxonomy, Thailand

## Abstract

**Background:**

Microsnails in the genus *Diplommatina* Benson, 1849 from eastern Thailand are revised, based on the collection of the Zoological Research Collection, Burapha University, Chonburi Province, Thailand and on recently-collected materials.

**New information:**

Five new species, *Diplommatina burapha* sp. n., *D. chadathongae* sp. n., *D. chantaburiensis* sp. n., *D. fusiformis* sp. n. and *D. khwantongae* sp. n., are described as new to science. The geographic distribution of these eastern species is presented.

## Introduction

Terrestrial microsnails in the genus *Diplommatina* Benson, 1849 show some of the greatest diversity and are distributed in southern, south-eastern and eastern Asia. At present, a total of 18 *Diplommatina* species have been reported from Thailand. Of the 18 described species, ten (55.55%) were recorded from the south and seven (38.9%) from the north, whereas only one species, *D. crispata khaochamaoensis* Panha et al., 1998, was described from Khaochamao National Park, Chantaburi Province (Maassen 2001, Panha 1996, Panha and Burch 1996, Panha and Burch 2001, Panha and Burch 2005, Panha et al. 1998, Tongkerd et al. 2013). This study is focused on a survey of microsnail diversity recorded in eastern Thailand since 2005 and focused on five species of the genus *Diplommatina* (*D. burapha* sp. n., *D. chadathongae* sp. n., *D. fusiformis* sp. n., *D. khwantongae* sp. n. and *D. khaowongensis*) as new to science, based on the external shell characters. The geographic distribution of these eastern Thai species is also presented.

## Materials and methods

**Figure 1.**
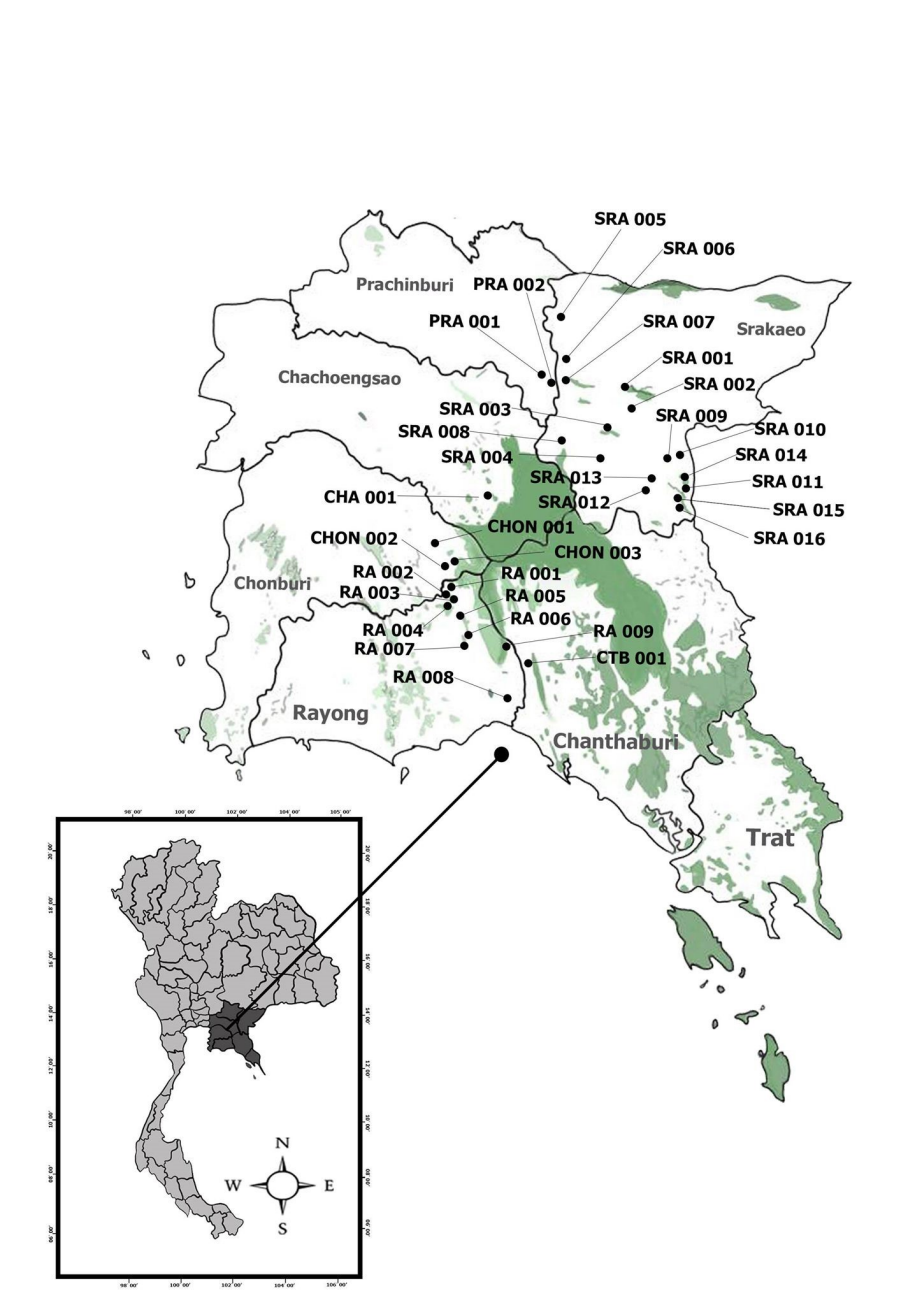
Collecting sites. The code indicated in the map follows the list in Table 1.

The specimens from the Zoological Research Collection of Burapha University (ZRCBUU) which have been collected since 2005 and recently obtained shell specimens from the field survey were studied. Shells were collected by hand searching and leaf litter sieving in several areas in eastern Thailand (Fig. 1 and Table 1). Empty shells were cleaned with tap water and a fine hairbrush and then air-dried. Only live specimens of one species, *Diplommatina khwantongae* were collected. Digital photographs of shells were obtained using a Canon MP-E 65 Macro lens camera. Shell measurements (in mm), including shell height (SH), shell width (SW), aperture height (AH) and aperture width (AW), were determined using ImageJ software. Taxonomic identification and shell terminology (e.g. whorl number and shell sculpture) of the specimens were recored according to Panha et al. (1998) and Tongkerd et al. (2013). Type specimens were deposited in the collection of the Zoological Research Collection of Burapha University (ZRCBUU), Chon Buri, Thailand.

## Taxon treatments

### Diplommatina (crispata) khaochamaoensis

Panha et al., 1998

A2FB9327-9270-521B-A29A-7C8690C5452D

#### Materials

- **Type status:** Other material. **Occurrence:** catalogNumber: ZRCBUU0019; recordedBy: Pongrat Dumrongrojwattana; individualCount: 8; lifeStage: adult; preparations: dry shell material; **Taxon:** scientificName: Diplommatina crispata khaochamaoensis; **Location:** country: Thailand; stateProvince: Rayong; locality: Khao Pratun, Khaochamao District; verbatimCoordinates: 13°07'36.9"N 101°35'56.6"E; georeferenceProtocol: GPS; **Identification:** identifiedBy: Pongrat Dumrongrojwattana; dateIdentified: 2004; **Event:** samplingProtocol: hand collecting; eventDate: 2004; habitat: limestone hills; **Record Level:** language: en; collectionCode: Mollusc; basisOfRecord: PreservedSpecimen
- **Type status:** Other material. **Occurrence:** catalogNumber: ZRCBUU0029; recordedBy: Pongrat Dumrongrojwattana; individualCount: 16; lifeStage: adult; preparations: dry shell material; **Taxon:** scientificName: Diplommatina crispata khaochamaoensis; **Location:** country: Thailand; stateProvince: Rayong; locality: Khao Pratun, Khaochamao District; verbatimCoordinates: 13°07'36.9"N 101°35'56.6"E; georeferenceProtocol: GPS; **Identification:** identifiedBy: Pongrat Dumrongrojwattana; dateIdentified: 2004; **Event:** samplingProtocol: hand collecting; eventDate: 2004; habitat: limestone hills; **Record Level:** language: en; collectionCode: Mollusc; basisOfRecord: PreservedSpecimen
- **Type status:** Other material. **Occurrence:** catalogNumber: ZRCBUU0042; recordedBy: Pongrat Dumrongrojwattana; individualCount: 15; lifeStage: adult; preparations: dry shell material; **Taxon:** scientificName: Diplommatina crispata khaochamaoensis; **Location:** country: Thailand; stateProvince: Chonburi; locality: Lublae Cave, Bothong District, Chonburi province; verbatimCoordinates: 13°09'09.0"N 101°35'52.1"E; georeferenceProtocol: GPS; **Identification:** identifiedBy: Pongrat Dumrongrojwattana; dateIdentified: 2004; **Event:** samplingProtocol: hand collecting; eventDate: 2004; habitat: limestone hills; **Record Level:** language: en; collectionCode: Mollusc; basisOfRecord: PreservedSpecimen
- **Type status:** Other material. **Occurrence:** catalogNumber: ZRCBUU0047; recordedBy: Pongrat Dumrongrojwattana; individualCount: 16; lifeStage: adult; preparations: dry shell material; **Taxon:** scientificName: Diplommatina crispata khaochamaoensis; **Location:** country: Thailand; stateProvince: Rayong; locality: Wat Tam Wattana Mongkol, Khaochamao District; verbatimCoordinates: 13°05'49.4"N 101°36'25.1"E; georeferenceProtocol: GPS; **Identification:** identifiedBy: Pongrat Dumrongrojwattana; dateIdentified: 2004; **Event:** samplingProtocol: hand collecting; eventDate: 2004; habitat: limestone hills; **Record Level:** language: en; collectionCode: Mollusc; basisOfRecord: PreservedSpecimen
- **Type status:** Other material. **Occurrence:** catalogNumber: ZRCBUU0051; recordedBy: Pongrat Dumrongrojwattana; individualCount: 10; lifeStage: adult; preparations: dry shell material; **Taxon:** scientificName: Diplommatina crispata khaochamaoensis; **Location:** country: Thailand; stateProvince: Chonburi; locality: Lublae Cave, Bothong District; verbatimCoordinates: 13°09'09.0"N 101°35'52.1"E; georeferenceProtocol: GPS; **Identification:** identifiedBy: Pongrat Dumrongrojwattana; dateIdentified: 2008; **Event:** samplingProtocol: hand collecting; eventDate: 2008; habitat: limestone hills; **Record Level:** language: en; collectionCode: Mollusc; basisOfRecord: PreservedSpecimen
- **Type status:** Other material. **Occurrence:** catalogNumber: ZRCBUU0079; recordedBy: Pongrat Dumrongrojwattana; individualCount: 10; lifeStage: adult; preparations: dry shell material; **Taxon:** scientificName: Diplommatina crispata khaochamaoensis; **Location:** country: Thailand; stateProvince: Rayong; locality: Khao Pratun, Khaochamao District; verbatimCoordinates: 13°07'36.9"N 101°35'56.6"E; georeferenceProtocol: GPS; **Identification:** identifiedBy: Pongrat Dumrongrojwattana; dateIdentified: 2008; **Event:** samplingProtocol: hand collecting; eventDate: 2008; habitat: limestone hills; **Record Level:** language: en; collectionCode: Mollusc; basisOfRecord: PreservedSpecimen
- **Type status:** Other material. **Occurrence:** catalogNumber: ZRCBUU0086; recordedBy: Pongrat Dumrongrojwattana; individualCount: 5; lifeStage: adult; preparations: dry shell material; **Taxon:** scientificName: Diplommatina crispata khaochamaoensis; **Location:** country: Thailand; stateProvince: Chonburi; locality: Lublae Cave, Bothong District; verbatimCoordinates: 13°09'09.0"N 101°35'52.1"E; georeferenceProtocol: GPS; **Identification:** identifiedBy: Pongrat Dumrongrojwattana; dateIdentified: 2015; **Event:** samplingProtocol: hand collecting; eventDate: 2015; habitat: limestone hills; **Record Level:** language: en; collectionCode: Mollusc; basisOfRecord: PreservedSpecimen
- **Type status:** Other material. **Occurrence:** catalogNumber: ZRCBUU0098; recordedBy: Pongrat Dumrongrojwattana; individualCount: 24; lifeStage: adult; preparations: dry shell material; **Taxon:** scientificName: Diplommatina crispata khaochamaoensis; **Location:** country: Thailand; stateProvince: Rayong; locality: Wat Rattanatrikoon, Khaochamao District; verbatimCoordinates: 12°56'53.1"N 101°46'37.1"E; georeferenceProtocol: GPS; **Identification:** identifiedBy: Pongrat Dumrongrojwattana; dateIdentified: 2013; **Event:** samplingProtocol: hand collecting; eventDate: 2013; habitat: limestone hills; **Record Level:** language: en; collectionCode: Mollusc; basisOfRecord: PreservedSpecimen
- **Type status:** Other material. **Occurrence:** catalogNumber: ZRCBUU0107; recordedBy: Pongrat Dumrongrojwattana; individualCount: 18; lifeStage: adult; preparations: dry shell material; **Taxon:** scientificName: Diplommatina crispata khaochamaoensis; **Location:** country: Thailand; stateProvince: Rayong; locality: Khao Pratun, Khaochamao District; verbatimCoordinates: 13°07'36.9"N 101°35'56.6"E; georeferenceProtocol: GPS; **Identification:** identifiedBy: Pongrat Dumrongrojwattana; dateIdentified: 2015; **Event:** samplingProtocol: hand collecting; eventDate: 2015; habitat: limestone hills; **Record Level:** language: en; collectionCode: Mollusc; basisOfRecord: PreservedSpecimen
- **Type status:** Other material. **Occurrence:** catalogNumber: ZRCBUU0108; recordedBy: Pongrat Dumrongrojwattana; individualCount: 5; lifeStage: adult; preparations: dry shell material; **Taxon:** scientificName: Diplommatina crispata khaochamaoensis; **Location:** country: Thailand; stateProvince: Chonburi; locality: Lublae Cave, Bothong District; verbatimCoordinates: 13°09'09.0"N 101°35'52.1"E; georeferenceProtocol: GPS; **Identification:** identifiedBy: Pongrat Dumrongrojwattana; dateIdentified: 2015; **Event:** samplingProtocol: hand collecting; eventDate: 2015; habitat: limestone hills; **Record Level:** language: en; collectionCode: Mollusc; basisOfRecord: PreservedSpecimen
- **Type status:** Other material. **Occurrence:** catalogNumber: ZRCBUU0109; recordedBy: Pongrat Dumrongrojwattana; individualCount: 5; lifeStage: adult; preparations: dry shell material; **Taxon:** scientificName: Diplommatina crispata khaochamaoensis; **Location:** country: Thailand; stateProvince: Rayong; locality: Khao Pratun, Khaochamao District; verbatimCoordinates: 13°07'36.9"N 101°35'56.6"E; georeferenceProtocol: GPS; **Identification:** identifiedBy: Pongrat Dumrongrojwattana; dateIdentified: 2015; **Event:** samplingProtocol: hand collecting; eventDate: 2015; habitat: limestone hills; **Record Level:** language: en; collectionCode: Mollusc; basisOfRecord: PreservedSpecimen
- **Type status:** Other material. **Occurrence:** catalogNumber: ZRCBUU0534; recordedBy: Pongrat Dumrongrojwattana; individualCount: 22; lifeStage: adult; preparations: dry shell material; **Taxon:** scientificName: Diplommatina crispata khaochamaoensis; **Location:** country: Thailand; stateProvince: Rayong; locality: Khao Pratun, Khaochamao District; verbatimCoordinates: 13°07'36.9"N 101°35'56.6"E; georeferenceProtocol: GPS; **Identification:** identifiedBy: Pongrat Dumrongrojwattana; dateIdentified: 2018; **Event:** samplingProtocol: hand collecting; eventDate: 2018; habitat: limestone hills; **Record Level:** language: en; collectionCode: Mollusc; basisOfRecord: PreservedSpecimen
- **Type status:** Other material. **Occurrence:** catalogNumber: ZRCBUU0619; recordedBy: Pongrat Dumrongrojwattana; individualCount: 22; lifeStage: adult; preparations: dry shell material; **Taxon:** scientificName: Diplommatina crispata khaochamaoensis; **Location:** country: Thailand; stateProvince: Chonburi; locality: Lublae Cave, Bothong District; verbatimCoordinates: 13°09'09.0"N 101°35'52.1"E; georeferenceProtocol: GPS; **Identification:** identifiedBy: Pongrat Dumrongrojwattana; dateIdentified: 2019; **Event:** samplingProtocol: hand collecting; eventDate: 2019; habitat: limestone hills; **Record Level:** language: en; collectionCode: Mollusc; basisOfRecord: PreservedSpecimen
- **Type status:** Other material. **Occurrence:** catalogNumber: ZRCBUU0739; recordedBy: Pongrat Dumrongrojwattana; individualCount: 11; lifeStage: adult; preparations: dry shell material; **Taxon:** scientificName: Diplommatina crispata khaochamaoensis; **Location:** country: Thailand; stateProvince: Rayong; locality: Wat Tam Wattana Mongkol, Khaochamao District; verbatimCoordinates: 13°05'49.4"N 101°36'25.1"E; georeferenceProtocol: GPS; **Identification:** identifiedBy: Pongrat Dumrongrojwattana; dateIdentified: 2020; **Event:** samplingProtocol: hand collecting; eventDate: 2020; habitat: limestone hills; **Record Level:** language: en; collectionCode: Mollusc; basisOfRecord: PreservedSpecimen
- **Type status:** Other material. **Occurrence:** catalogNumber: ZRCBUU0740; recordedBy: Piyaporn Muenrit; individualCount: 11; lifeStage: adult; preparations: dry shell material; **Taxon:** scientificName: Diplommatina crispata khaochamaoensis; **Location:** country: Thailand; stateProvince: Rayong; locality: Pradoo Cave, Khaochamao District; verbatimCoordinates: 13°05'40.8"N 101°36'25.1"E; georeferenceProtocol: GPS; **Identification:** identifiedBy: Pongrat Dumrongrojwattana; dateIdentified: 2020; **Event:** samplingProtocol: hand collecting; eventDate: 2020; habitat: limestone hills; **Record Level:** language: en; collectionCode: Mollusc; basisOfRecord: PreservedSpecimen
- **Type status:** Other material. **Occurrence:** catalogNumber: ZRCBUU0741; recordedBy: Sasicha Techama; individualCount: 11; lifeStage: adult; preparations: dry shell material; **Taxon:** scientificName: Diplommatina crispata khaochamaoensis; **Location:** country: Thailand; stateProvince: Rayong; locality: Khao Pratun, Khaochamao District; verbatimCoordinates: 13°07'36.9"N 101°35'56.6"E; georeferenceProtocol: GPS; **Identification:** identifiedBy: Pongrat Dumrongrojwattana; dateIdentified: 2020; **Event:** samplingProtocol: hand collecting; eventDate: 2020; habitat: limestone hills; **Record Level:** language: en; collectionCode: Mollusc; basisOfRecord: PreservedSpecimen
- **Type status:** Other material. **Occurrence:** catalogNumber: ZRCBUU0742; recordedBy: Onchira Saenkamon; individualCount: 11; lifeStage: adult; preparations: dry shell material; **Taxon:** scientificName: Diplommatina crispata khaochamaoensis; **Location:** country: Thailand; stateProvince: Rayong; locality: Khao Pratun, Khaochamao District; verbatimCoordinates: 13°07'36.9"N 101°35'56.6"E; georeferenceProtocol: GPS; **Identification:** identifiedBy: Pongrat Dumrongrojwattana; dateIdentified: 2020; **Event:** samplingProtocol: hand collecting; eventDate: 2020; habitat: limestone hills; **Record Level:** language: en; collectionCode: Mollusc; basisOfRecord: PreservedSpecimen
- **Type status:** Other material. **Occurrence:** catalogNumber: ZRCBUU0744; recordedBy: Sirilak Bu-On; individualCount: 24; lifeStage: adult; preparations: dry shell material; **Taxon:** scientificName: Diplommatina crispata khaochamaoensis; **Location:** country: Thailand; stateProvince: Rayong; locality: Khao Pratun, Khaochamao District; verbatimCoordinates: 13°07'36.9"N 101°35'56.6"E; georeferenceProtocol: GPS; **Identification:** identifiedBy: Pongrat Dumrongrojwattana; dateIdentified: 2020; **Event:** samplingProtocol: hand collecting; eventDate: 2020; habitat: limestone hills; **Record Level:** language: en; collectionCode: Mollusc; basisOfRecord: PreservedSpecimen
- **Type status:** Other material. **Occurrence:** catalogNumber: ZRCBUU0758; recordedBy: Noppadon Wiboonpuech; individualCount: 11; lifeStage: adult; preparations: dry shell material; **Taxon:** scientificName: Diplommatina crispata khaochamaoensis; **Location:** country: Thailand; stateProvince: Rayong; locality: Khao Pratun, Khaochamao District; verbatimCoordinates: 13°07'36.9"N 101°35'56.6"E; georeferenceProtocol: GPS; **Identification:** identifiedBy: Pongrat Dumrongrojwattana; dateIdentified: 2020; **Event:** samplingProtocol: hand collecting; eventDate: 2020; habitat: limestone hills; **Record Level:** language: en; collectionCode: Mollusc; basisOfRecord: PreservedSpecimen

#### Distribution

Limestone areas in Chonburi and Rayong provinces

### Diplommatina burapha

Dumrongrojwattana, Kamtuptim & Wongkamhaeng 2020 sp. n.

A2E6CA09-8B19-5151-9EB3-26A78E1A0F48

B98B3418-9B9F-4F5E-AD09-C6DB5AEBE2AA

#### Materials

- **Type status:** Holotype. **Occurrence:** catalogNumber: ZRCBUU-0747; recordedBy: Pongrat Dumrongrojwattana; individualCount: 1; lifeStage: adult; preparations: dry shell material; disposition: in collection; **Taxon:** scientificName: Diplommatina burapha; **Location:** country: Thailand; stateProvince: Srakeo; locality: Wat Tam Khaochan, Khaochakan District; verbatimElevation: 120-130 m; verbatimCoordinates: 13°34'43.2"N 102°05'34.8"E; decimalLatitude: 13.525939; decimalLongitude: 102.076315; georeferenceProtocol: GPS; **Identification:** identifiedBy: Pongrat Dumrongrojwattana; dateIdentified: 2020; **Event:** samplingProtocol: hand collecting; eventDate: 2020; habitat: The wall of limestone hills that is located in a temple which is surrounded by variety of plants including the member of Euphorbiaceae, Leguminosae and Orchidaceae.; **Record Level:** language: en; collectionCode: Mollusc; basisOfRecord: PreservedSpecimen
- **Type status:** Paratype. **Occurrence:** catalogNumber: ZRCBUU 0748; recordedBy: Pongrat Dumrongrojwattana; individualCount: 26; lifeStage: adult; preparations: dry shell material; **Taxon:** scientificName: Diplommatina burapha; **Location:** country: Thailand; stateProvince: Srakeo; locality: Wat Tam Khaochan, Khaochakan District; verbatimCoordinates: 13°34'43.2"N 102°05'34.8"E; georeferenceProtocol: GPS; **Identification:** identifiedBy: Pongrat Dumrongrojwattana; dateIdentified: 2020; **Event:** samplingProtocol: hand collecting; eventDate: 2020; habitat: The wall of three limestone hills that is located in a temple which issurrounded by variety of plants including the members of Euphorbiaceae, Leguminosae and Orchidaceae.; **Record Level:** language: en; collectionCode: Mollusc; basisOfRecord: PreservedSpecimen

#### Description

**Figure 2.**
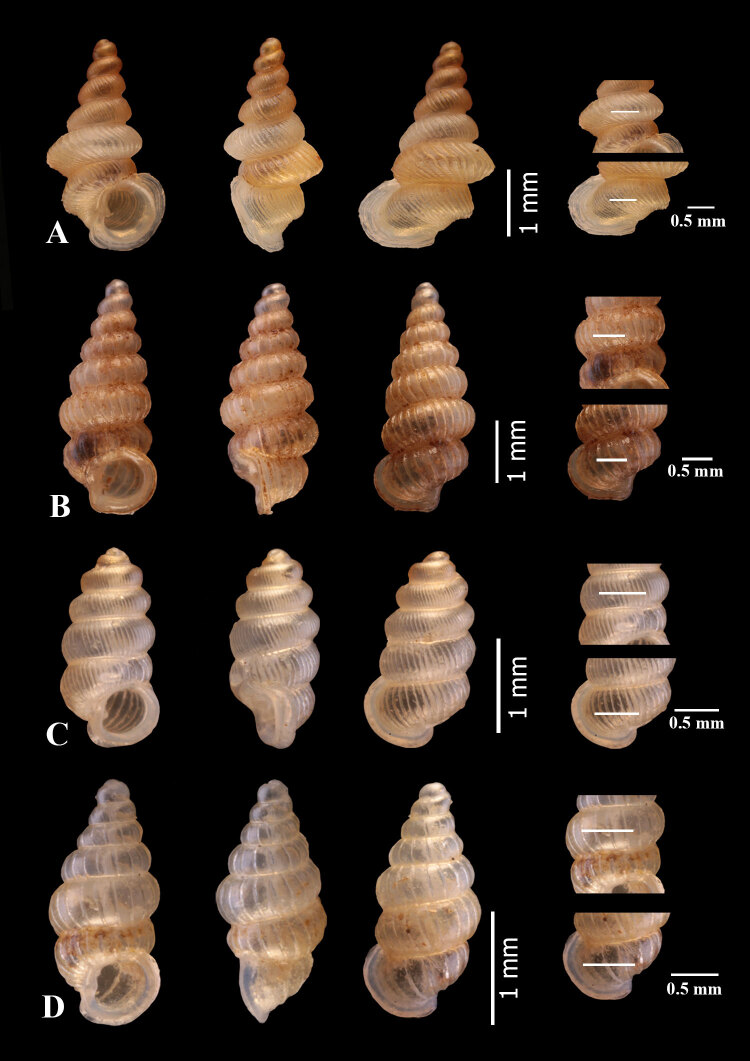
Four *Diplommatina species* from eastern Thailand. A) *Diplommatina crispata khaochamaoensis* Panha et al., 1998, B) *D. burapha* sp. n., C) *D. chadathongae* sp. nov. and D) *D. chantaburiensis* sp. n. Each species shows a frontal view, lateral view, dorsal view and the number of ribs per 0.5 mm on the penultimate whorl and body whorl, respectively.

**Holotype**: Shell height 3.59 mm, shell width 1.91 mm. Aperture height 0.93 mm, aperture width 1.86 mm. Shell width/shell height ratio = 0.53. Aperture width/aperture height ratio = 1.15 (Fig. 2A).

**Paratypes**: Shell height 3.59-4.35 mm (4.11 ± 0.11 mm) shell width 1.91-2.35 mm (2.21 ± 0.07 mm). Aperture height 0.93-1.19 mm (1.08 ± 0.06 mm). Aperture width 1.07-1.47 mm (1.39 ± 0.07 mm). Shell width/shell height ratio = 0.54 ± 0.02. Aperture width/aperture height ratio = 1.29 ± 0.05.

**Shell** minute dextral, elongated narrow, turreted with 7 ¾ round whorls that increase regularly in size and diameter until the last whorl; **protoconch** orange, smooth, consisting of 1 ¼ whorls, covered with minute pits; **teleoconch** light orange, consists of about 5 ¾ whorls that increase regularly in size and diameter until the last whorl; **spires** shouldered; **sculpture** consists of rather even and rather widely-spaced radial ribs with about 8 ribs/mm on the penultimate whorl and about 6 ribs/mm on the body whorl, the discernible spiral striae between the ribspresent; **sutures** deep; **umbilicus** closed; **aperture** round; **peristome** is thickened and expanded and doubled; **columellar** lamella rather bluntly rounded (Fig. 4).

#### Diagnosis

Shell minute, dextral, turreted, translucent, whitish; protoconch smooth; teleoconch sculpture of widely-spaced radial ribs with discernible spiral striae between the ribs, Aperture rounded, columellar lamella well developed, peristome doubled, thickened and expanded.

#### Etymology

We named this new species “*burapha*,” which means “eastern.” This refers to the type locality, which is located in the east and is also in honour of Burapha University, the workplace of the authors.

#### Distribution

This species is known only from the type locality.

#### Taxon discussion

The size of the shell, shape of the shell and protoconch of *D. burapha* sp. n. are similar to *D. doichiangdao* Panha & Burch, 1998 from Doi Chiang Dao, northern Thailand and Laotian species, *D. belonis* Möllendorff, 1900. The spire of *D. burapha* sp. n. is relatively broader than that of those two species. The enlarged penultimate whorl and the body whorl of *D. burapha* sp. n. are relatively similar in diameter, while the last whorls of both *D. doichiangdao* and *D. belonis* are smaller in diameter than the enlarged penultimate whorl. This new species columellar is poorly developed, while well developed in *D. doichiangdao* and *D. belonis*.

### Diplommatina chadathongae

Kamtuptim, Dumrongrojwattana & Wongkamhaeng 2020 sp. n.

16AF65E0-732C-5168-ABFC-D1B333EBD608

4ACB0FCD-80A2-44C1-A213-3734A0F3B5F0

#### Materials

- **Type status:** Holotype. **Occurrence:** catalogNumber: ZRCBUU-0092; recordedBy: Pongrat Dumrongrojwattana; individualCount: 1; lifeStage: adult; preparations: dry shell material; **Taxon:** scientificName: Diplommatina chadathongae; **Location:** country: Thailand; stateProvince: Chanthaburi; locality: Wat Khaowongkotrujiwongsaram, Kanghaengmaeo District; verbatimCoordinates: 12°53'11.4"N 101°49'06.2"E; georeferenceProtocol: GPS; **Identification:** identifiedBy: Pongrat Dumrongrojwattana; dateIdentified: 2012; **Event:** samplingProtocol: hand collecting; eventDate: 2020; habitat: limestone hills; **Record Level:** language: en; collectionCode: Mollusc; basisOfRecord: PreservedSpecimen
- **Type status:** Paratype. **Occurrence:** catalogNumber: ZRCBUU-0093; recordedBy: Pongrat Dumrongrojwattana; individualCount: 29; lifeStage: adult; preparations: dry shell material; **Taxon:** scientificName: Diplommatina chadathongae; **Location:** country: Thailand; stateProvince: Chanthaburi; locality: Wat Tam Khaochan, Khaochakan District; verbatimCoordinates: 12°53'11.4"N 101°49'06.2"E; georeferenceProtocol: GPS; **Identification:** identifiedBy: Pongrat Dumrongrojwattana; dateIdentified: 2012; **Event:** samplingProtocol: hand collecting; eventDate: 2020; habitat: limestone hills; **Record Level:** language: en; collectionCode: Mollusc; basisOfRecord: PreservedSpecimen

#### Description

**Holotype.** Shell height 1.61 mm, shell width 0.78 mm. Aperture height 0.50 mm, aperture width 0.60 mm. Shell width/shell height ratio = 0.49. Aperture width/aperture height ratio = 1.21 (Fig. 2C).

**Paratypes (6 shells).** Shell height 1.93-2.07 mm (1.98 ± 0.05 mm), shell width 0.84-0.93 mm (0.88 ± 0.03 mm). Aperture height 0.51-0.63 mm (0.58 ± 0.05 mm). Aperture width 0.59-0.67 mm (0.63 ± 0.02 mm). Shell width/shell height ratio = 0.45 ± 0.01. Aperture width/aperture height ratio = 1.09 ± 0.07.

**Shell** minute dextral, cylindrical, translucent, whitish, with about 5 ¾ whorls that increase regularly in size and diameter until the last whorl; **protoconch** orange, smooth, consisting of 1 ½ whorls; **teleoconch** light orange, consisting of about 4 ¼ keeled whorls; **sculpture** consists of strong, rather narrowly-spaced radial ribs which are about 20 ribs/mm on the penultimate whorl and about 14 ribs/mm on the body whorl; **sutures** well impressed; **umbilicus** closed; **aperture** round; **peristome** thickened and expanded and doubled; **columellar** lamella well developed and directed anteriorly (Fig. 4).

#### Diagnosis

Shell minute, dextral, cylindrical, translucent, whitish, teleoconch sculpture of narrowly-spaced axial ribs, whorls aperture rounded, columellar lamella well developed, peristome thickened, expanded and doubled.

#### Etymology

We named this new species “*chadathongae*” in memory of the second author's beloved mother, Ms. Wiang Chadathong.

#### Distribution

Only known from the type locality.

#### Taxon discussion

*Diplommatina chadathongae* sp. n. is another one of the Thai smallest *Diplommatina* species (average shell height = 1.69 ± 0.04 mm) and same size as *D. krabiensis* Panha & Burch, 1998 (shell height of type specimens are 1.6-1.7 mm and average shell height = 1.69 ± 0.04 mm). This new species differs from *D. krabiensis* by its dextral, cylindrical, narrowly-fine radial ribs, while the smallest species is sinistral, shell fusiform or ovate with widely-spaced radial ribs. This new species differs from *Diplommatina miriensis* Godwin-Austen, 1917 from Nepal by its shell being more slender and columellar lamella well developed.

### Diplommatina chantaburiensis

Dumrongrojwattana, Kamtuptim & Wongkamhaeng 2020 sp. n.

BEE18D52-FE05-5835-8823-1D56004680F1

2573D215-407A-42C5-AD42-7E365477CA7D

#### Materials

- **Type status:** Holotype. **Occurrence:** catalogNumber: ZRCBUU-0090; recordedBy: Pongrat Dumrongrojwattana; individualCount: 1; lifeStage: adult; preparations: dry shell material; **Taxon:** scientificName: Diplommatina chantaburiensis; **Location:** country: Thailand; stateProvince: Chanthaburi; locality: Wat Khao Wong Kot, Kanghaengmaeo District; verbatimCoordinates: 12°52'47.3"N 101°49'13.8"E; georeferenceProtocol: GPS; **Identification:** identifiedBy: Pongrat Dumrongrojwattana; dateIdentified: 2012; **Event:** samplingProtocol: hand collecting; eventDate: 2020; habitat: limestone hills; **Record Level:** language: en; collectionCode: Mollusc; basisOfRecord: PreservedSpecimen
- **Type status:** Paratype. **Occurrence:** catalogNumber: ZRCBUU-0091; recordedBy: Pongrat Dumrongrojwattana; individualCount: 29; lifeStage: adult; preparations: dry shell material; **Taxon:** scientificName: Diplommatina chantaburiensis; **Location:** country: Thailand; stateProvince: Chanthaburi; locality: Wat Khao Wong Kot, Kanghaengmaeo District; verbatimCoordinates: 12°52'47.3"N 101°49'13.8"E; georeferenceProtocol: GPS; **Identification:** identifiedBy: Pongrat Dumrongrojwattana; dateIdentified: 2012; **Event:** samplingProtocol: hand collecting; eventDate: 2020; habitat: limestone hills; **Record Level:** language: en; collectionCode: Mollusc; basisOfRecord: PreservedSpecimen

#### Description

**Holotype.** Shell height 3.56 mm, shell width 1.64 mm. Aperture height 1.03 mm, aperture width 1.23 mm. Shell width/shell height ratio = 0.46. Aperture width/aperture height ratio = 1.19 (Fig. 2D).

**Paratypes (7 shells).** Shell height 2.56-2.85 mm (2.67 ± 0.10 mm) shell width 1.13-1.28 mm (1.24 ± 0.05 mm). Aperture height 0.73-0.85 mm (0.77 ± 0.06 mm). Aperture width 0.73-1.01 mm (0.93 ± 0.08 mm). Shell width/shell height ratio = 0.46 ± 0.02. Aperture width/aperture height ratio = 1.20 ± 0.13.**Shell** minute dextral, fusiform, translucent, whitish, with about 7 whorls that increase regularly in both size and diameter until the last whorl which is slightly smaller in diameter than the penultimate whorl; **protoconch** large, whitish, smooth, surface cover with minute pits, consisting of 1 ¼ whorls; **teleoconch** about 5 ¼ whorls, whitish; **sculpture** consists of thin, widely-spaced radial ribs which about 9 ribs/mm on the penultimate whorl and about 6 ribs/mm on the body whorl, discernible spiral striae between the ribs absent; **sutures** deeply impressed; **umbilicus** closed; **aperture** rounded; **peristome** thicked and expanded; **columellar** lamella is relatively small (Fig. 4).

#### Diagnosis

Shell minute, dextral, translucent, whitish, protoconch large, teleoconch sculpture consisting of widely-spaced axial ribs, spiral striae between the ribs absent; aperture rounded, columellar lamella relatively small, peristome thickened and expanded.

#### Etymology

The species name “*chantaburiensis*” refers to Chantaburi, the Province of the type locality.

#### Distribution

Only known from the type locality.

#### Taxon discussion

*Diplommatina chantaburiensis* sp. n. is very similar to *D. fusiformis* sp. n. Nevertheless, *D. chantaburiensis* sp. n. has larger protoconch, shell more slender and no spiral striae between the radial ribs. This new species is also similar to the southern species *D. hidagai* Panha, 1998, but differs by its prominent protoconch, more widely-radial ribs and columellar lamella is poorly developed.

### Diplommatina fusiformis

Dumrongrojwattana, Kamtuptim & Wongkamhaeng 2020 sp. n.

51EDA5AD-1843-5208-B481-68F2EB761887

92F4C2C9-E2CC-43EA-825A-5052282885ED

#### Materials

- **Type status:** Holotype. **Occurrence:** catalogNumber: ZRCBUU-0105; recordedBy: Pongrat Dumrongrojwattana; individualCount: 1; lifeStage: adult; preparations: dry shell material; **Taxon:** scientificName: Diplommatina fusiformis; **Location:** country: Thailand; stateProvince: Rayong; locality: Wat Rattanatrikoon, Khaochamao District; verbatimCoordinates: 12°56'53.1"N 101°46'37.1"E; georeferenceProtocol: GPS; **Identification:** identifiedBy: Pongrat Dumrongrojwattana; dateIdentified: 2015; **Event:** samplingProtocol: hand collecting; eventDate: 2020; habitat: limestone hills; **Record Level:** language: en; collectionCode: Mollusc; basisOfRecord: PreservedSpecimen
- **Type status:** Paratype. **Occurrence:** catalogNumber: ZRCBUU-0104; recordedBy: Pongrat Dumrongrojwattana; individualCount: 18; lifeStage: adult; preparations: dry shell material; **Taxon:** scientificName: Diplommatina fusiformis; **Location:** country: Thailand; stateProvince: Rayong; locality: Wat Rattanatrikoon, Khaochamao District; verbatimCoordinates: 12°56'53.1"N 101°46'37.1"E; georeferenceProtocol: GPS; **Identification:** identifiedBy: Pongrat Dumrongrojwattana; dateIdentified: 2015; **Event:** samplingProtocol: hand collecting; eventDate: 2020; habitat: limestone hills; **Record Level:** language: en; collectionCode: Mollusc; basisOfRecord: PreservedSpecimen

#### Description

**Figure 3.**
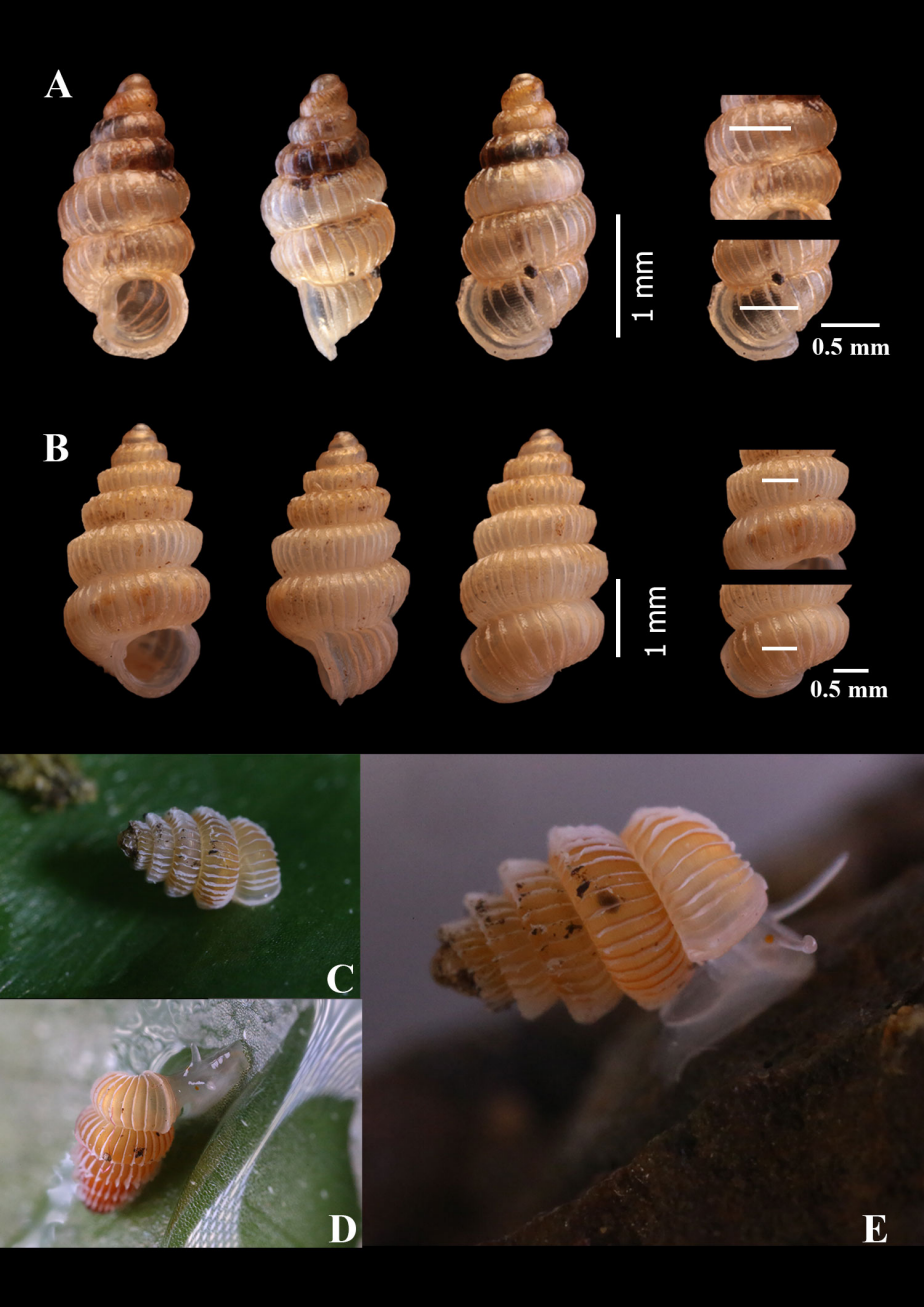
Two Diplommatina species from eastern Thailand. A) D. fusiformis spec. nov., B-E) D. khwantongae spec. nov., B) Holotype, C-D) Living snail. (Photos by Ms. Rattanawadee Tekavong). Each species shows a frontal view, lateral view, dorsal view and the number of ribs per 0.5 mm on the penultimate whorl and body whorl, respectively.

**Holotype.** Shell height 2.66 mm, shell width 1.22 mm. Aperture height 0.76 mm, aperture width 0.94 mm. Shell width/shell height ratio = 0.46. Aperture width/aperture height ratio = 1.23 (Fig. 3A).

**Paratypes (5 shells).** Shell height 2.66-2.74 mm (2.65 ± 0.07 mm) shell width 1.21-1.29 mm (1.25 ± 0.03 mm). Aperture height 0.76-0.87 mm (0.78 ± 0.06 mm). Aperture width 0.82-0.98 mm (0.91 ± 0.06 mm). Shell width/shell height ratio = 0.47 ± 0.02. Aperture width/aperture height ratio = 1.18 ± 0.10.

**Figure 4.**
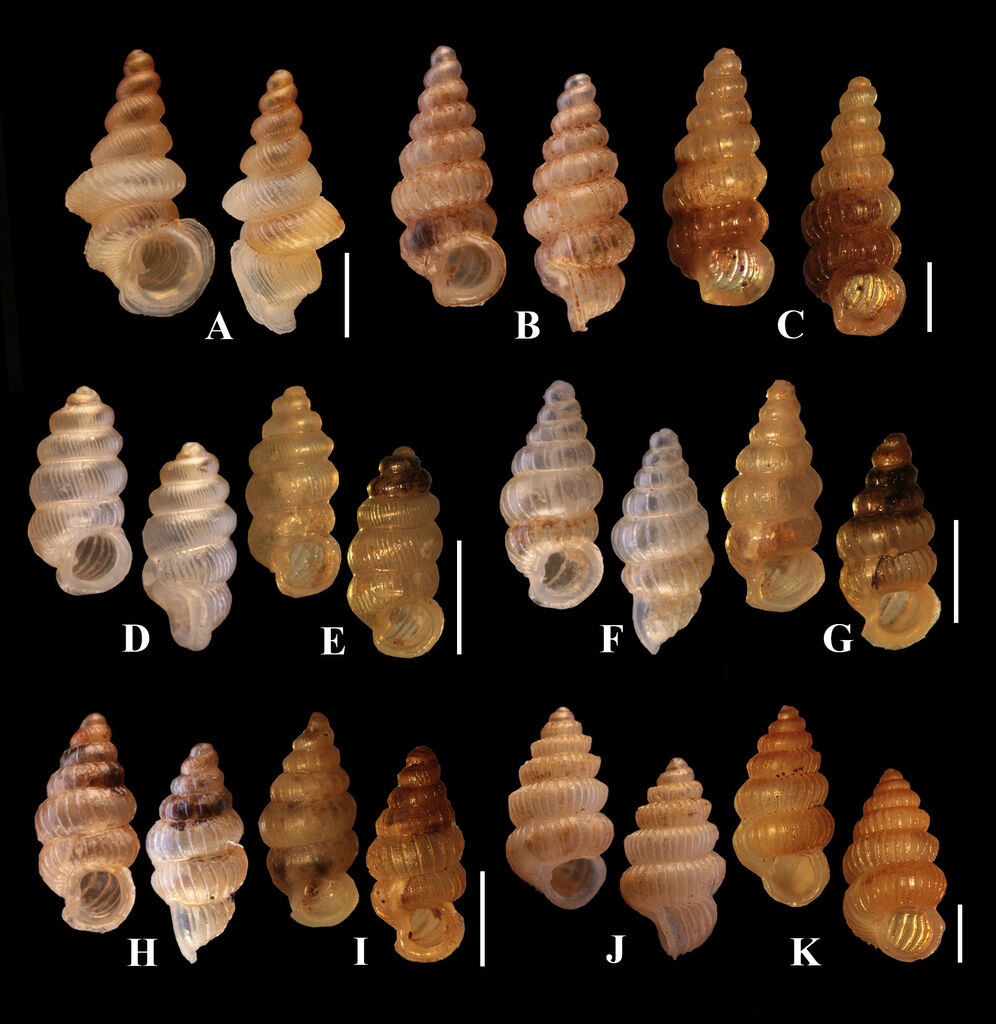
Synopsis of eastern diplommatind species. A) *Diplommatina crispata khaochamaoensis*, B-C) Holotype and paratypes of *D. burapha* sp. n., D-E) Holotype and paratypes of *D. chadathongae* sp. n., F-G) Holotype and paratypes of *D. chantaburiensis* sp. n., H-I) Holotype and paratypes of *D. fusiformis* sp. n. and J-K) Holotype and paratypes of *D. khwantongae* sp. n. Scale bar = 1 mm.

**Description. Shell** minute dextral, fusiform, translucent, light orange, with about 6 ½ whorls that increase regularly in size and diameter until the last whorl which is slightly smaller in diameter than the penultimate whorl; **protoconch** orange, smooth, consisting of 1 ¼ whorls; **teleoconch** light orange, about 5 ¼ whorls; **sculpture** consists of thin, widely-spaced radial ribs with discernible spiral striae between the ribs; there are about 10 ribs/mm on the penultimate whorl and about 8 ribs/mm on the body whorl; **sutures** deep; **umbilicus** closed; **aperture** rounded, peristome thickened and expanded; **columellar** lamella relatively small (Fig. 4) .

#### Diagnosis

**Diagnosis.** Shell minute, dextral, translucent light orange, teleoconch sculpture consisting of widely-spaced axial ribs with spiral striae between the ribs; aperture rounded, columellar lamella relatively small, peristome, thickened and expanded.

#### Etymology

We named this new species “*fusiformis*” based on its fusiform shell shape.

#### Distribution

Only known from the type locality.

#### Taxon discussion

*Diplommatina fusiformis* sp. n. is similar to the southern species, *D. hidagai* Panha, 1998, but it differs by its more widely-ribbed shell, its more slender spire and its less prominent columellar lamella.

### Diplommatina khwantongae

Dumrongrojwattana, Kamtuptim & Wongkamhaeng 2020 sp. n.

D6207A03-7664-5B49-BB45-E5D13EA1C0FF

4311F0B5-9CA7-460F-9637-1C11F9BEDD86

#### Materials

- **Type status:** Holotype. **Occurrence:** catalogNumber: ZRCBUU-0190; recordedBy: Kanita Khwantong; individualCount: 1; lifeStage: adult; preparations: dry shell material; **Taxon:** scientificName: Diplommatina khwantongae; **Location:** country: Thailand; stateProvince: Chonburi; locality: Lub Lae Cave, Bothong district, Chonburi Province; verbatimCoordinates: 13°09'09.9"N 101°35'52.4"E; georeferenceProtocol: GPS; **Identification:** identifiedBy: Kanita Khwantong; dateIdentified: 2015; **Event:** samplingProtocol: hand collecting; eventDate: 2020; habitat: limestone hills; **Record Level:** language: en; collectionCode: Mollusc; basisOfRecord: PreservedSpecimen
- **Type status:** Paratype. **Occurrence:** catalogNumber: ZRCBUU-0102; recordedBy: Kanita Khwantong; individualCount: 5; lifeStage: adult; preparations: dry shell material; **Taxon:** scientificName: Diplommatina khwantongae; **Location:** country: Thailand; stateProvince: Chonburi; locality: Lub Lae Cave, Bothong district, Chonburi Province; verbatimCoordinates: 13°09'09.9"N 101°35'52.4"E; georeferenceProtocol: GPS; **Identification:** identifiedBy: Kanita Khwantong; dateIdentified: 2015; **Event:** samplingProtocol: hand collecting; eventDate: 2020; habitat: limestone hills; **Record Level:** language: en; collectionCode: Mollusc; basisOfRecord: PreservedSpecimen
- **Type status:** Paratype. **Occurrence:** catalogNumber: ZRCBUU-0757; recordedBy: Rattanawadee Tekavong; individualCount: 5; lifeStage: adult; preparations: dry shell material; **Taxon:** scientificName: Diplommatina khwantongae; **Location:** country: Thailand; stateProvince: Chonburi; locality: Lub Lae Cave, Bothong district, Chonburi Province; verbatimCoordinates: 13°09'09.9"N 101°35'52.4"E; georeferenceProtocol: GPS; **Identification:** identifiedBy: Pongrat Dumrongroiwattana; dateIdentified: 2015; **Event:** samplingProtocol: hand collecting; eventDate: 2020; year: 2020; month: 7; day: 11; habitat: limestone hills; **Record Level:** language: en; collectionCode: Mollusc; basisOfRecord: PreservedSpecimen
- **Type status:** Other material. **Occurrence:** catalogNumber: ZRCBUU-0759; occurrenceRemarks: Three live specimens were collected and reared in the laboratory for few months. All specimens were preserved in ethanol.; recordedBy: Rattanawadee Tekavong; individualCount: 3; lifeStage: adult; **Taxon:** scientificName: Diplommatina khwantongae; **Location:** country: Thailand; stateProvince: Chonburi; locality: Lub Lae Cave, Bothong district, Chonburi Province; verbatimCoordinates: 13°09'09.9"N 101°35'52.4"E; georeferenceProtocol: GPS; **Identification:** identifiedBy: Pongrat Dumrongroiwattana; dateIdentified: 2015; **Event:** samplingProtocol: hand collecting; eventDate: 2020; year: 2020; month: 7; day: 11; habitat: limestone hills; **Record Level:** language: en; collectionCode: Mollusc; basisOfRecord: PreservedSpecimen

#### Description

**Holotype**- Shell height 3.59 mm, shell width 1.91 mm. Aperture height 0.93 mm, aperture width 1.86 mm. Shell width/shell height ratio = 0.53. Aperture width/aperture height ratio = 1.15. (Fig. 3B-E).

**Paratypes: (6 shells).** Shell height 1.68-1.76 mm (1.69 ± 0.04 mm), shell width 0.78-0.81 mm (0.81 ± 0.04 mm). Aperture height 0.46-0.58 mm (0.53 ± 0.04 mm). Aperture width 0.53-0.61 mm (0.59 ± 0.03 mm). Shell width/shell height ratio = 0.48 ± 0.03. Aperture width/aperture height ratio = 1.12 ± 0.07.

**Shell** minute dextral, cylindrical, light orange, with about 7 whorls that increase regularly in size and diameter until the last whorl; **protoconch** orange, smooth, consisting of 1 ¼ whorls, covered with minute pits; **teleoconch** light orange, consisting of about 5 ¾ whorls; **spires** shouldered; **sculpture** consists of rather even and widely-spaced thin radial ribs with discernible spiral striae between the ribs; there are about 8 ribs/mm on the penultimate whorl and about 6 ribs/mm on the body whorl; **sutures** deep; **umbilicus** closed; **aperture** round; **peristome** thickened and expanded and doubled; **columellar lamella** rather bluntly rounded.

**Animal.** Living animals had a greyish body, head, tentacles and foot, with an orange eye located at the base of each tentacle (Fig. 3C-E). The operculum was round and corneous. Snails were found living in leaf litter and plant debris in the limestone hills. Due to the small population of each species, the live specimens of all species found in this study are hard to find. More observation and further study on anatomy are needed.

#### Diagnosis

Shell minute, dextral, cylindrical, light orange, teleoconch sculpture of widely-spaced and high radial ribs, aperture rounded, columellar lamella well developed, peristome thickened and expanded.

#### Etymology

We named this new species “*khwantongae*” in memory of Ms. Kanita Khwantong, who made the first discovery of this new species.

#### Distribution

This species is known only from the limestone hills in Chonburi and Rayong Provinces.

#### Taxon discussion

The shell shape of *Diplommatina* is generally fusiform or tower-shaped with mostly low and strong radial ribs, which are formed as tubular projections in some species and mostly rounded or angular periphery whorls. For example, the shell of *D. hidagai* is fusiform covered with low and strong radial ribs or the shell of *D. nimannandhi* Panha et al., 2002 has tower-shaped, radial ribs forming as semi-tubular peripheral projections. In this new species, *Diplommatina khwantongae* sp. n. is a very distinct species of epitoniid snail due to its cylindrical, thin radial ribs and shouldered whorls.

## Discussion

**Figure 5.**
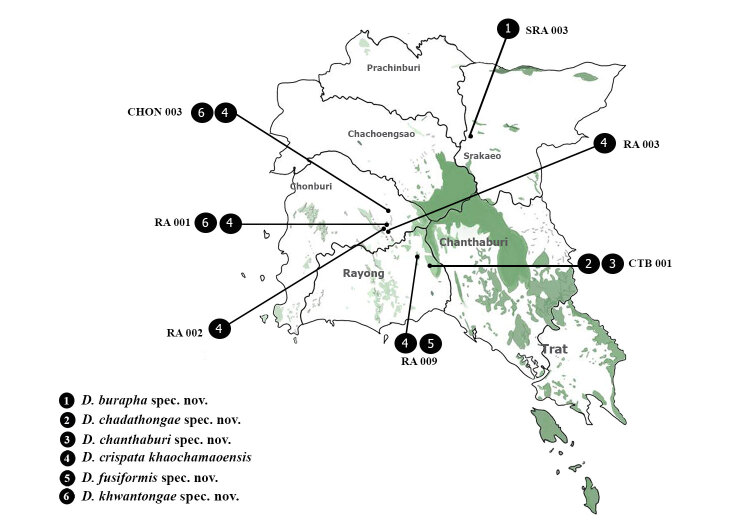
Distribution of *Diplommatina* spp. in Eastern Thailand. Code of location shown in Table 1.

In Thailand, a total of 18 *Diplommatina* species have been reported (Inkhavilay et al. 2019,Panha and Burch 2005, Tongkerd et al. 2013). From that, there is only one species, *Diplommatina crispata khaochamaoensis* recorded in eastern Thailand. This work increases the number of species reported in the eastern part of Thailand to five species and makes a total of 22 species of the *Diplommatina* in Thailand. These data imply that eastern Thailand might be a hot spot for the *Diplommatina.* Previous research on this microsnails' group reveals the high endemism in almost every species due to their ecology, living on the limestone hill that limits their dispersal ability (Panha and Burch 2005). In this study, the species described also show high endemism except for the *Diplommatina crispata khaochamaoensis*, which occurred in almost all study sites. (Fig. 5)

## Supplementary Material

XML Treatment for Diplommatina (crispata) khaochamaoensis

XML Treatment for Diplommatina burapha

XML Treatment for Diplommatina chadathongae

XML Treatment for Diplommatina chantaburiensis

XML Treatment for Diplommatina fusiformis

XML Treatment for Diplommatina khwantongae

## Figures and Tables

**Table 1.** List and coordination of collecting sites.

**Province**	**Code**	**Location**	Coordination (Lat. Long)
Chonburi	CHON 001	Khaocha-ang Pluangtong	13°11'56.4"N 101°34'47.0"E
	CHON 002	Khao Mhe Cave	13°09'04.0"N 101°35'35.0"E
	CHON 003	Lublae Cave	13°09'09.0"N 101°35'52.1"E
Rayong	RA 001	Khao Pratun	13°07'36.9"N 101°35'56.6"E
	RA 002	Wat Tam Wattana Mongkol	13°05'49.4"N 101°36'25.1"E
	RA 003	Pradoo Cave	13°05'40.8"N 101°36'25.1"E
	RA 004	Khao Loi Cave	13°03'26.8"N 101°36'26.2"E
	RA 005	Wat Tam Khaobote	13°02'14.5"N 101°38'04.4"E
	RA 006	Wat Tam Suwannaphupha	12°59'20.2"N 101°39'36.0"E
	RA 007	Samnaksong Tam Naeramitr	12°58'13.7"N 101°39'45.7"E
	RA 008	Wat Tam Rakangtong	12°45'48.3"N 101°47'47.2"E
	RA 009	Wat Rattanatrikoon	12°56'53.1"N 101°46'37.1"E
Chantaburi	CTB 001	Wat Tam Khaowong	14°35'15.5"N 101°20'33.5"E
	CHA 001	Wat Khao Tam Rat	13°23'30.3"N 101°44'39.6"E
Srakeo	SRA 001	Wat Tam Khao Chakan	13°39'38.0"N 102°05'02.8"E
	SRA 002	Samnaksong Phuming	13°38'52.5"N 102°05'52.4"E
	SRA 003	Wat Tam Khaochan	13°34'43.2"N 102°05'34.8"E
	SRA 004	Wat Petpananikom	13°29'17.9"N 102°04'48.3"E
	SRA 005	Wat Khao Singto	13°59'22.4"N 102°00'29.3"E
	SRA 006	Wat Tam Maka	13°47'49.6"N 101°57'54.3"E
	SRA 007	Wat Khao Sampung	13°39'07.2"N 101°56'53.5"E
	SRA 008	Wat Saitong	13°32'48.1"N 101°57'17.4"E
	SRA 009	Khao Phaphueng	13°28'27.1"N 102°17'00.9"E
	SRA 010	Khao Saraphee	13°28'36.8"N 102°19'06.3"E
	SRA 011	Khao Laeum	13°24'23.3"N 102°18'05.3"E
	SRA 012	Wat Tam Khao Phuheep	13°23'12.5"N 102°15'13.0"E
	SRA 013	Wat Sabthavorn	13°24'19.9"N 102°16'29.6"E
	SRA 014	Tam Pet Phoe Tong	13°24'49.2"N 102°19'31.3"E
	SRA 015	Tam Nam Pra Shiva	13°19'15.2"N 102°19'40.1"E
	SRA 016	Tam Saengtien	13°18'57.2"N 102°19'57.2"E
Pracheenburi	PRA 001	Wat Khao Teppitak	13°39'37.4"N 101°54'52.5"E
	PRA 002	Khao Tam Namtip	13°39'19.2"N 101°55'44.1"E
